# Apolipoprotein CIII Overexpressing Mice Are Predisposed to Diet-Induced Hepatic Steatosis and Hepatic Insulin Resistance

**DOI:** 10.1002/hep.24571

**Published:** 2011-08-19

**Authors:** Hui-Young Lee, Andreas L Birkenfeld, Francois R Jornayvaz, Michael J Jurczak, Shoichi Kanda, Violeta Popov, David W Frederick, Dongyan Zhang, Blas Guigni, Kalyani G Bharadwaj, Cheol Soo Choi, Ira J Goldberg, Jae-Hak Park, Kitt F Petersen, Varman T Samuel, Gerald I Shulman

**Affiliations:** 1 Department of Internal Medicine, Yale University School of MedicineNew Haven, CT; 2 Department of Cellular & Molecular Physiology, Yale University School of MedicineNew Haven, CT; 3 Howard Hughes Medical Institute, Yale University School of MedicineNew Haven, CT; 4 Department of Medicine, Columbia UniversityNew York, NY; 5 Department of Laboratory Animal Medicine, College of Veterinary Medicine, Seoul National UniversitySeoul, Korea

## Abstract

Nonalcoholic fatty liver disease (NAFLD) and insulin resistance have recently been found to be associated with increased plasma concentrations of apolipoprotein CIII (APOC3) in humans carrying single nucleotide polymorphisms within the insulin response element of the APOC3 gene. To examine whether increased expression of APOC3 would predispose mice to NAFLD and hepatic insulin resistance, human APOC3 overexpressing (ApoC3Tg) mice were metabolically phenotyped following either a regular chow or high-fat diet (HFD). After HFD feeding, ApoC3Tg mice had increased hepatic triglyceride accumulation, which was associated with cellular ballooning and inflammatory changes. ApoC3Tg mice also manifested severe hepatic insulin resistance assessed by a hyperinsulinemic-euglycemic clamp, which could mostly be attributed to increased hepatic diacylglycerol content, protein kinase C-ε activation, and decreased insulin-stimulated Akt2 activity. Increased hepatic triglyceride content in the HFD-fed ApoC3Tg mice could be attributed to a ≍70% increase in hepatic triglyceride uptake and ≍50% reduction hepatic triglyceride secretion. *Conclusion:* These data demonstrate that increase plasma APOC3 concentrations predispose mice to diet-induced NAFLD and hepatic insulin resistance. (Hepatology 2011;54:1650-1660)

Hepatic insulin resistance associated with nonalcoholic fatty liver disease (NAFLD) is a major factor contributing to the pathogenesis of type 2 diabetes.^1^-^3^ Hepatic fat accumulation is the result of an imbalance between lipid delivery and *de novo* lipogenesis and lipid disposal either by oxidation or export in very low density lipoproteins (VLDL). Apolipoprotein CIII (APOC3) has been shown to be an important factor in regulating plasma triglyceride concentrations; in both rodents and human subjects increased expression of APOC3 is associated with higher plasma triglyceride levels and a null mutation with reduced triglyceride levels.^4^, ^5^ APOC3 predominantly affects VLDL-triglyceride metabolism and greater APOC3 production can increase VLDL-triglyceride production and reduce hepatic clearance of triglyceride rich lipoproteins (TGRLs).^6^ APOC3 stimulates VLDL synthesis and secretion in cultured hepatocytes^7^ and elevated circulating APOC3 levels correlate with VLDL production^8^ and higher postprandial hyperlipidemia in humans.^9^ Furthermore, APOC3 expression was proinflammatory in endothelial cells^10^ and adipose tissue.^11^ Elevated circulating APOC3 levels are associated with many diseases including metabolic syndrome,^12^ coronary artery disease,^5^ and insulin resistance^9^, ^13^ both in humans and animal models.

Recently, we found that common rs2854116 [T-455C] and rs2854117 [C-482T] single nucleotide polymorphisms in an insulin-response element of the promoter region in the APOC3 gene were associated with increased plasma APOC3 concentrations and an increased prevalence of NAFLD and whole-body insulin resistance in healthy lean men.^9^ These results suggest that increased plasma APOC3 concentrations may predispose lean individuals to NAFLD and hepatic insulin resistance, although the underlying mechanisms for hepatic lipid accumulation and insulin resistance are unclear.

In order to examine these questions we examined: (1) whether transgenic mice with hepatic overexpression of human APOC3 (ApoC3Tg) are prone to develop nonalcoholic steatohepatitis (NASH); (2) whether NAFLD is associated with hepatic insulin resistance in this model; 3) the underlying mechanism by which these mice develop NAFLD; and (4) the cellular mechanisms by which NAFLD in these mice leads to hepatic insulin resistance.

## Materials and Methods

### Animals

The ApoC3Tg mice, under the control of its native promoter in the C57BL/6J background,^4^, ^6^ were further backcrossed with female C57BL/6J mice over five generations in the Yale animal facility. Male ApoC3Tg mice and age-matched littermate control male mice (WT) were studied at the age of 4 to 8 months. Mice were individually housed under controlled temperature (23°C) and lighting (12/12-hour light/dark) with free access to water and fed ad libitum on regular chow (RC, 2018S, Harlan Teklad) and a high-fat diet (HFD, 55% calories from fat, TD93075, Harlan Teklad). Body composition was assessed by ^1^H magnetic resonance spectroscopy (Bruker BioSpin). Whole-body energy metabolism including VO_2_, VCO_2_, energy expenditure, locomoter activity, and food intake were measured for 72 hours by indirect calorimeter (CLAMS, Columbus Instrument). The study was conducted at the NIH-Yale Mouse Metabolic Phenotyping Center. All procedures were approved by the Yale University Animal Care and Use Committee.

### Plasma Parameters

Blood samples were taken from the tail vein after an overnight fast. Plasma triglyceride and nonesterified fatty acid concentrations were determined using standard commercial kits according to the manufacturer's instructions (Diagnostic Chemicals and Wako Chemicals, respectively). Plasma insulin and adiponectin levels were measured by radioimmunoassays (Linco, Billerica, MA). Plasma glucose was measured with a glucose analyzer (Beckman Coulter, Brea, CA). Human APOC3 concentrations were measured with Cobas Mira plus (Roche Diagnostic, Indianapolis, IN). Mouse cytokine array (RayBio) was conducted as described in the manufacturer's description.

### Liver Histology

Liver sections for histology were obtained after overnight fasting, fixed in 10% formalin, and stained with hematoxylin-eosin or Masson trichrome. Histology was read by two independent pathologists blinded to experimental design and treatment groups. Briefly, steatosis (0-3), lobular inflammation (0-3), ballooning (0-3), and fibrosis (0-4) were scored separately on a scale of 0-4 as described.^14^, ^15^ NAFLD activity score is expressed as the sum of each scoring.^15^

### Hyperinsulinemic-Euglycemic Clamp Study

The mice were maintained on an HFD for ≍2 months. After an overnight fast, [3-^3^H]-glucose (high-performance liquid chromatography [HPLC] purified; PerkinElmer, Waltham, MA) was infused at a rate of 0.05 μCi/min for 2 hours to assess a rate of basal glucose turnover, followed by a 140-minute hyperinsulinemic-euglycemic clamp with a primed/continuous infusion of human insulin (31.5 mU/kg prime for 3 minutes, 4.5 mU/(kg-min) infusion; Novo Nordisk, Copenhagen, DK) as described.^16^, ^17^ Rates of basal and insulin-stimulated whole-body glucose fluxes and tissue glucose uptake were determined by bolus injection of 10 μCi of 2-deoxy-D-[1-^14^C] glucose (PerkinElmer).

### Tissue Lipid Measurements

Tissue triglyceride was extracted using the method of Bligh and Dyer^18^ and measured using a DCL Triglyceride Reagent (Diagnostic Chemicals). Hepatic ceramide species and hepatic cytosolic DAG species were measured after overnight fasting using liquid chromatography and tandem mass spectrometry as described.^19^ Total DAG and ceramide are expressed as the sum of individual species.

### Membrane Translocation of Protein Kinase C-ε (PKCε) and Phosphorylated AKT

PKCε membrane translocation in the liver protein extracts after overnight fasting and insulin phosphorylation of AKT^Ser473^ were assessed both after overnight fasting and after the hyperinsulinemic euglycemic clamp study using the methods described.^20^, ^21^ Images were analyzed and quantified with ImageJ (NIH). Membrane translocation of PKCε was expressed as the ratio of membrane to cytosol bands.

### Tissue Lipid Clearance and Uptake

Plasma lipid clearance and tissue uptake were assessed using mouse [^3^H]-labeled triolein and endogenously dual labeled chylomicrons (CM), including [^3^H]-retinyl ester and [^14^C]-triglyceride. Animals fed HFD for ≍3 months were cannulated (jugular vein) 7 days before the injection day. After collection of overnight fasting blood samples, a bolus injection of Liposyn (20%, 0.75 mL/kg, Abbott Laboratories, North Chicago, IL) conjugated with 10 μCi of [9,10-^3^H(N)]-triolein was administered over 1 minute through the jugular vein. Blood samples were collected at 2.5, 5, 7.5, 10, 15, 20, 30, and 60 minutes from tail vein. Endogenously dual-radiolabeled CM were prepared as described.^22^ After collection of blood at 0 minutes, 2 × 10^5^ dpm of [^14^C]-triglyceride-CM and 6 × 10^5^ dpm of [^3^H]-RE-CM were injected. Blood was collected at 2.5, 5, 10, and 15 minutes after injection. Tissue and plasma organic phase were extracted using the method of Bligh and Dyer^18^ and ^3^H/^14^C radioactivity was measured by beta-counter and the plasma and tissue triglyceride concentration was determined using a DCL Triglyceride Reagent (Diagnostic Chemicals). Tissue-specific triglyceride uptake rates [mg/(kg tissue-min)] were calculated by the following equation: Triglyceride uptake rate [mg/(kg tissue-min)] = plasma triglyceride (mg/ml) × {tissue radio-activity [dpm/(kg tissue-min)]/area under curve (AUC) of plasma radioactivity (dpm/ml)}.

### Liver Triglyceride Production

Mice were maintained on RC or HFD for 3 months. After an overnight fast, plasma triglyceride levels were assayed prior to (0 hours) and 1, 2, 3, and 4 hours after poloxamer407 injection using an enzymatic method (Diagnostic Chemicals). The VLDL-triglyceride production rate was calculated by the increase in plasma triglyceride level from baseline to 4 hours after 1 g/kg of poloxamer407 intraperitoneal injection and the data were expressed as micromoles of triglyceride produced per hour per kg of body weight. Plasma apolipoprotein B (apoB) levels were determined by western blotting using rabbit polyclonal antibody (Abcam) in 4%-12% gradient gel (Invitrogen). The same membrane was stained with Coomassie-blue to show equal loading.

### Intraperitoneal Glucose Tolerance Test (IPGTT)

Weight-matched mice were maintained on an HFD for 6 weeks. After an overnight fast mice were weighed and received a bolus intraperitoneal injection of glucose (1 g/kg). Blood samples were obtained from a tail vein at baseline (0) and 15, 30, 45, 60, 90, and 120 minutes after glucose challenge for determination of plasma glucose and insulin concentrations.

### Reverse-Transcription Polymerase Chain Reaction (RT-PCR)

Tissue samples were obtained from mice maintained on RC or HFD for 3 months. The detailed methods, gene expression data, and sequences of PCR primers used are described in Supporting Table 2.

### Statistics

All values are expressed as mean ± SEM. The significance of the differences in mean values among two groups was evaluated by two-tailed unpaired Student's *t* tests. More than three groups were evaluated by analysis of variance (ANOVA) followed by post-hoc analysis using Bonferroni's Multiple Comparison Test. *P* < 0.05 was considered significant.

## Results

### APOC3 Overexpression Promotes the Development of Diet-Induced Hepatic Steatosis and NAFLD in Mice

**Fig. 1:**
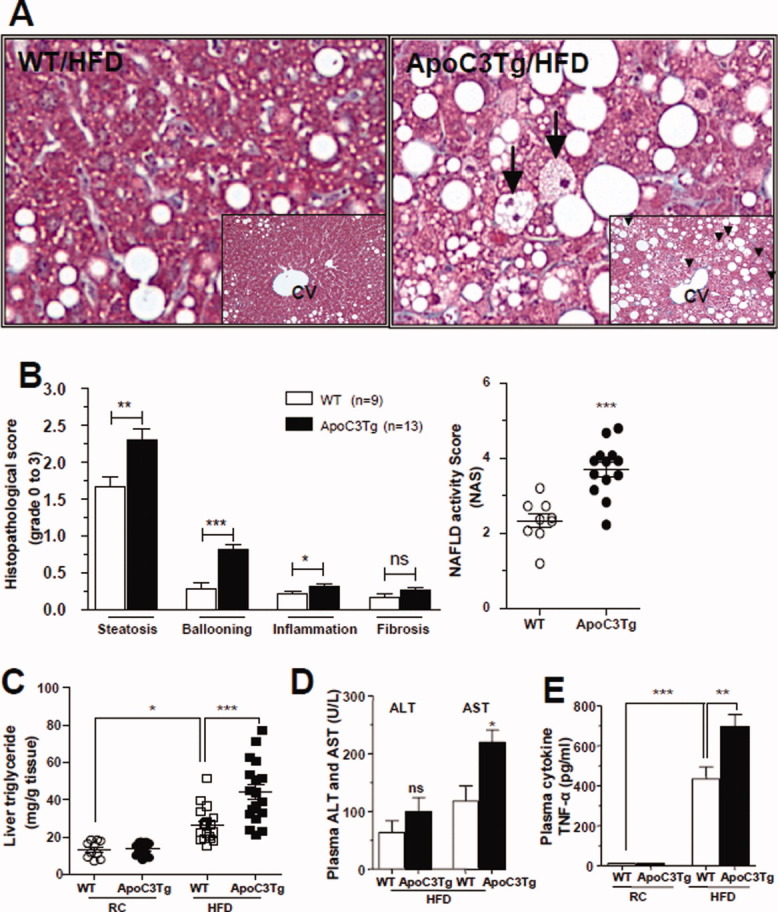
APOC3 promote diet-induced hepatic steatosis and liver damage. Animals fed an RC and HFD for 2-3 months and fasted overnight prior to taking liver and blood samples. (A) Representative histological section stained with Masson-trichrome staining, examined in ×400 light microscopy field after HFD feeding for 2-3 months (insert ×200 magnifications). Arrow indicates ballooned hepatocyte. (B) Histological scoring of NAFLD in liver section after HFD feeding for 3 months. (C) Triglyceride level in the liver of WT and ApoC3Tg mice after RC and 2-3 months of HFD feeding (n > 10). (D) Plasma ALT and AST concentration after 3 months of HFD feeding (n = 4). (E) Plasma TNF-α concentration in the WT and ApoC3Tg mice after RC and 3 months of HFD feeding (n = 3, equal amounts of 4 mice plasma were pooled into each sample). Data are expressed as mean ± SEM. *P < 0.05, **P < 0.01, and ***P < 0.001 by Student's *t* test for (B,D) and by ANOVA with post-hoc analysis for (C,E).

Increases in plasma human APOC3 were associated with significantly higher levels of plasma triglyceride in ApoC3Tg mice (Table 1). Both on RC and HFD, body weight, body composition, whole-body energy metabolism (VO_2_, VCO_2_, respiratory quotient, energy expenditure, food intake, and locomotor activity) were identical (Table 1) between WT and ApoC3Tg mice. Both groups showed identical increases in body weight and there were no differences in body composition while consuming a HFD (Table 1). Fasting plasma fatty acid concentrations were ≍70% higher in ApoC3Tg mice, compared with WT mice (Table 1). The ApoC3Tg mice fed the HFD for ≍3 months exhibited more severe hepatic steatosis with lipid accumulation around the Zone 3 area (Fig. 1A) than the WT, accompanied by ≍60% increased liver triglyceride levels (Fig. 1C), which was similar between WT and ApoC3Tg mice on RC (Supporting Fig. 1). Ballooned cells, characterized by ≍2 times the size of adjacent hepatocytes and pyknotic nucleus, were increased in ApoC3Tg mice compared with WT mice after HFD feeding (Fig. 1A). The increased liver triglyceride content in the ApoC3Tg mice was associated with an ≍80% increase in serum aspartate aminotransferase levels and a tendency for higher serum alanine aminotransferase levels (Fig. 1D). Fasting plasma proinflammatory cytokines were markedly increased both in WT and ApoC3Tg mice fed the HFD compared with RC fed mice and there were further increases in plasma tumor necrosis factor alpha (TNF-α) and interferon gamma (INF-γ) (both ≍70%) in ApoC3Tg mice compared with the WT mice after HFD feeding (Fig. 1E, Supporting Fig. 2). We applied a system of histological scoring designed for use in humans to systematically compare the differences in histology between the two groups. The NAFLD activity score (NAS) of ApoC3Tg mice was ≍4 (Fig. 1B) after HFD feeding, and NAS of ≥5 has been correlated with a diagnosis of NASH.^15^ In contrast, there were no significant differences in plasma concentrations of interleukin (IL)-1β, IL-10, IL-12p70, IL-8, and IL-6 levels (Supporting Fig. 2A) or hepatic nuclear factor kappaB (NF-κB) p65, IkappaBα, or c-Jun N-terminal kinase (JNK) phosphorylation (Supporting Fig. 2A,B) between genotypes fed the HFD.

**Table 1:** Basal Characterization for Animals

	RC		HFD	
		
	WT	ApoC3Tg	WT	ApoC3Tg
Body weight (g)	29.6 ± 0.3 (n=8)	29.7 ± 0.4 (n=8)	39.5 ± 1.2† (n=8)	40.7 ± 1.1† (n=8)
Liver weight (g)	0.93 ± 0.03 (n=4)	1.0 ± 0.06 (n=4)	1.72 ± 0.12† (n=6)	2.18 ± 0.18† (n=6)
Epididymal WAT weight (g)	0.28 ± 0.05 (n=4)	0.23 ± 0.03 (n=4)	2.9 ± 0.23† (n=6)	3.1 ± 0.26† (n=6)
Body fat (%)	8.98 ± 0.9 (n=8)	9.7 ± 0.74 (n=8)	25.98 ± 1.3† (n=8)	26.31 ± 1.1† (n=8)
Lean body mass (%)	74.45 ± 0.85 (n=8)	74.29 ± 0.71 (n=8)	62.76 ± 1.43† (n=8)	63.35 ± 0.98† (n=8)
Fasting glucose (mg/dL)	118.3 ± 9.4 (n=6)	122.7 ± 11.4 (n=6)	156.4 ± 6.7† (n=8)	149.1 ± 10.9† (n=8)
Fasting insulin (μU/mL)	8.3 ± 2.0 (n=6)	11.0 ± 1.5 (n=6)	30.3 ± 6.1† (n=8)	37.3 ± 9.9† (n=8)
Fasting NEFA (mg/dL)	1.07 ± 0.11 (n=4)	1.80 ± 0.15** (n=4)	0.64 ± 0.05† (n=6)	0.97 ± 0.08*,† (n=6)
Fasting plasma triglyceride (mg/dL)	92.3 ± 6.8 (n=8)	1342 ± 221*** (n=8)	88.1 ± 14.1 (n=12)	617 ± 96.3***,† (n=12)
Human plasma APOC3 (mg/dL)	ND	209.2 ± 54.1 (n=8)	ND	106.4 ± 18.9† (n=8)
Fasting plasma adiponectin (μg/mL)	39.9 ± 3.5 (n=4)	31.4 ± 2.3 (n=4)	26.2 ± 1.9† (n=4)	31.9 ± 4.0 (n=4)
Whole body VO_2_ [Kcal/(kg-hr)]	3077 ± 52 (n=8)	3039 ± 56 (n=8)	2775 ± 77† (n=8)	2743 ± 52† (n=8)
Whole body VCO_2_ [Kcal/(kg-hr)]	2662 ± 60 (n=8)	2627 ± 62 (n=8)	2121 ± 57† (n=8)	2112 ± 44† (n=8)
Food intake [Kcal/(kg-hr)]	17.8 ± 1.2 (n=8)	17.5 ± 1.3 (n=8)	12.67 ± 0.52† (n=8)	13.72 ± 1.28† (n=8)
Respiratory quotient	0.86 ± 0.01 (n=8)	0.86 ± 0.01 (n=8)	0.76 ± 0.01† (n=8)	0.77 ± 0.01† (n=8)
Activity (counts)	143.6 ± 14.5 (n=8)	176 ± 25.5 (n=8)	91.0 ± 21.9 (n=8)	110.1 ± 17.4 (n=8)

WT and ApoC3Tg mice fed a RC and high fat diet for 2-3 month and fasted overnight prior to experiments.

Data are expressed as mean values ± SEM.

* *P* < 0.05,

** *P* < 0.01,

*** *P* < 0.001 by unpaired Student's *t* test compared to WT versus ApoC3Tg group.

† *P* < 0.05 by unpaired Student's *t* test compared to RC versus HFD in each group.

ND, not detected. NEFA, nonesterified fatty acid.

### ApoC3Tg Mice Manifest Hepatic Insulin Resistance

**Fig. 2:**
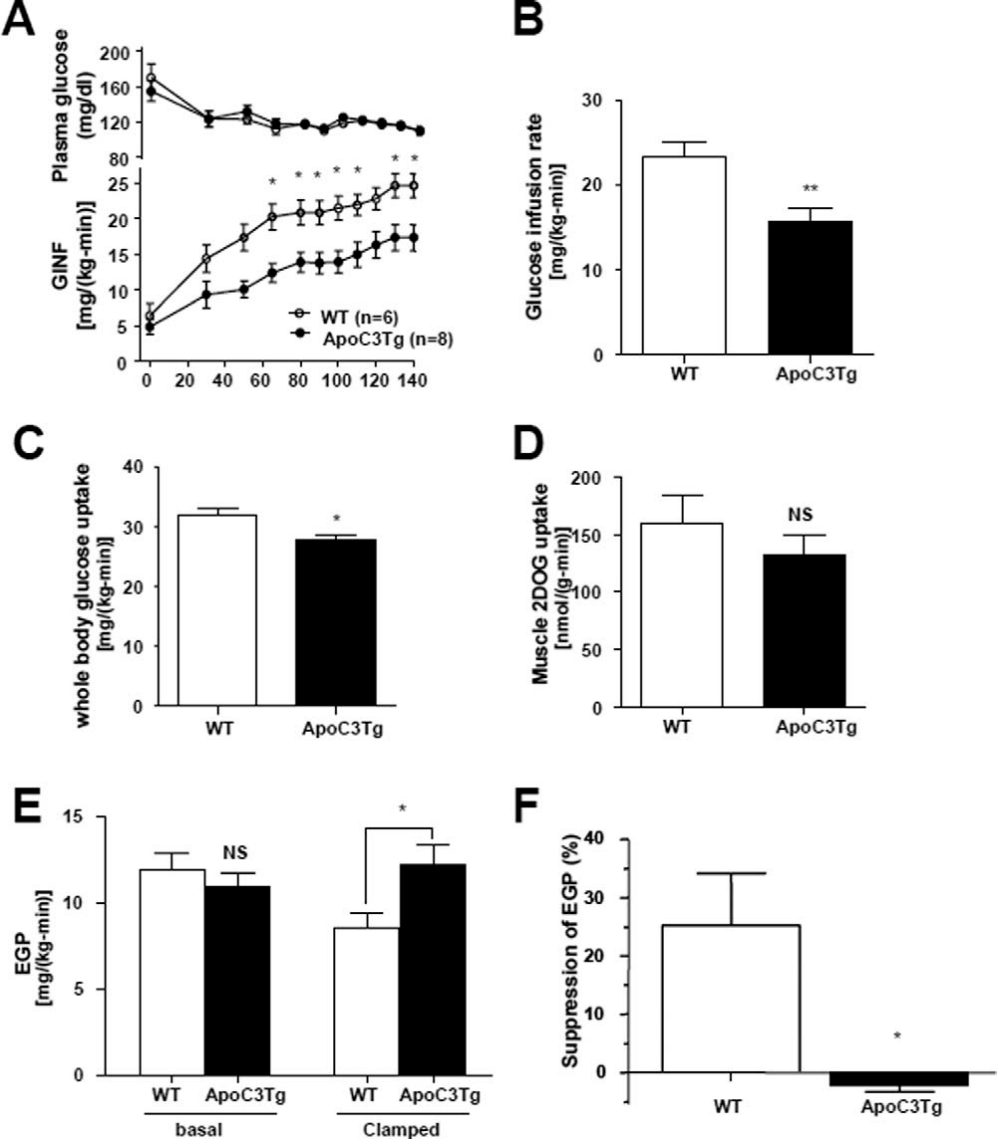
Hepatic steatosis in ApoC3Tg mice is associated with hepatic insulin resistance. (A) Plasma glucose concentration and glucose infusion rate in WT (n = 6) and ApoC3Tg (n = 7) mice during hyperinsulinemic-euglycemic clamp study following 2 months of HFD. (B,C) Glucose infusion rate and whole-body glucose uptake rate during steady status. (D) Insulin stimulated muscle 2-deoxy-D-[1-^14^C] glucose (2DOG) uptake in gastrocnemius muscle. (E) Basal EGP and insulin suppressed (clamped) EGP and (F) percentage of suppression during the hyperinsulinemic-euglycemic clamp study. Data are expressed as mean ± SEM. *P < 0.05, **P < 0.01, and ***P < 0.001 by Student's *t* test except (A) (2-way ANOVA with post-hoc analysis).

**Fig. 3:**
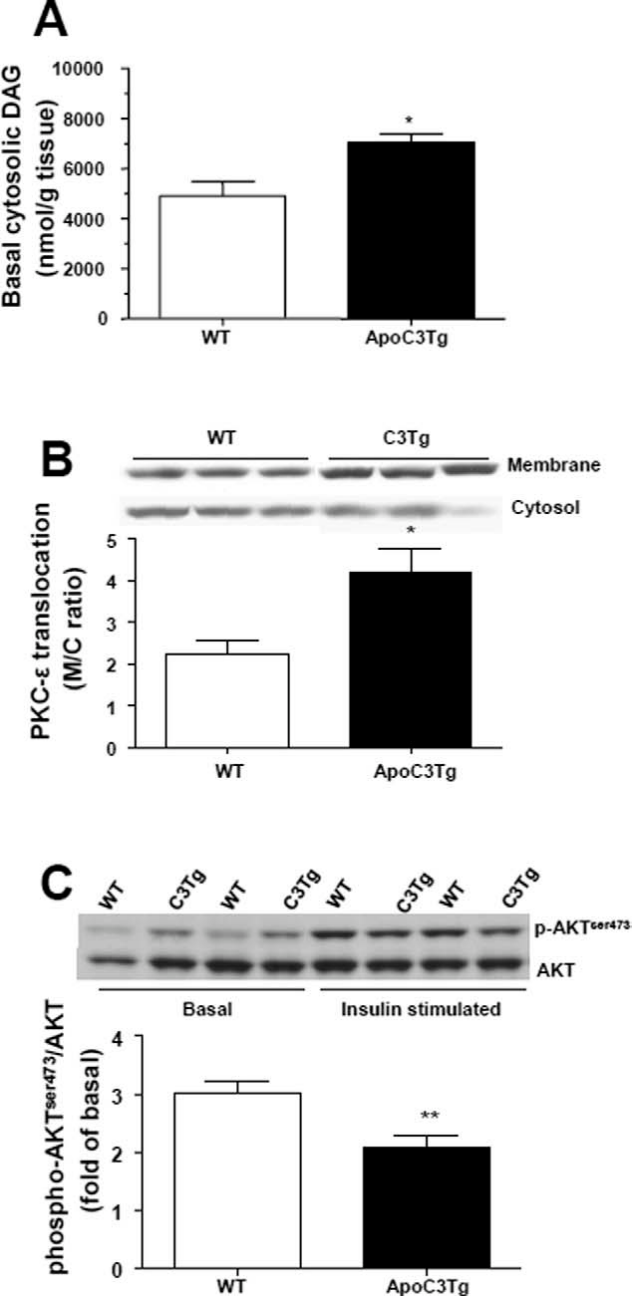
Hepatic insulin resistance in the ApoC3Tg mice is associated with increased hepatic diacylglycerol content and PKCε activation. Animals fed an RC and HFD for 2 months and fasted overnight prior to experiments. (A) Intracellular cytosolic diacylglycerol after overnight fasting. (B) Membrane translocation of PKCε was analyzed in the liver of WT and ApoC3Tg mice after hyperinsulinemic-euglycemic clamp study. (C) Akt phosphorylation on Ser^473^ was analyzed in the liver of overnight-fasted and hyperinsulinemic-euglycemic clamped WT and ApoC3Tg mice after 2 months of HFD feeding. Data are expressed as mean ± SEM. (A), n = 6; (B), n = 4; (C), n = 6-7 per group. *P < 0.05, **P < 0.01 by Student's *t* test.

In order to assess the tissue-specific contributions of liver, skeletal muscle, and adipose tissue to whole-body insulin resistance in the HFD-fed ApoC3Tg mice, we performed hyperinsulinemic-euglycemic clamp studies combined with ^3^H/^14^C-labeled glucose infusions.^16^, ^17^ Overnight fasting plasma glucose and insulin concentrations were similar between the two groups (Table 1) as were basal rates of endogenous glucose production (EGP) (Fig. 2E). During the hyperinsulinemic-euglycemic clamp the glucose infusion rates required to maintain euglycemia (Fig. 2A) in the ApoC3Tg mice were 34% lower than those of WT mice (Fig. 2B), indicating that the ApoC3Tg mice were more insulin resistant than the age-weight-matched WT mice. The ApoC3Tg mice displayed marked hepatic insulin resistance as reflected by the lack of suppression of EGP during the hyperinsulinemic-euglycemic clamp compared with the WT mice (Fig. 2E,F). Rates of insulin-stimulated whole-body glucose uptake were also slightly decreased by 13% in the ApoC3Tg mice compared with the WT mice (Fig. 3C), whereas muscle 2-deoxy-glucose uptake was not significantly different (Fig. 2D). Fasting plasma fatty acid concentrations decreased in a similar fashion during the hyperinsulinemic-euglycemic clamp study in both groups (% suppression: WT, 42% ± 7%; ApoC3Tg, 45% ± 6%), indicating that adipose tissue insulin responsiveness was similar between the two groups. Plasma insulin concentrations were similar between WT and ApoC3Tg mice during the clamp study (Supporting Table 1).

### ApoC3Tg Mice Have Increased Hepatic DAG Content, PKCε Activation, and Hepatic Insulin Resistance

Increased hepatic triglyceride content and hepatic insulin resistance was associated with ≍45% increase in hepatic cytosolic DAG content in the liver from ApoC3Tg mice compared with the WT mice fed the HFD (Fig. 3A), whereas hepatic ceramide content was not different between genotypes (Supporting Fig. 3C,D). Furthermore, this increase in liver DAG content was associated with an ≍90% increase in the membrane/cytosol ratio of PKCε (Fig. 3B), reflecting an increase in PKCε activity, and a 32% reduction in insulin-stimulated AKT2 activity in the livers of ApoC3Tg mice compared with WT mice (Fig. 3C). Taken together, these data are consistent with the hypothesis that increased plasma levels of APOC3 predispose animals to NAFLD and hepatic insulin resistance, which in turn can be attributed to DAG-induced PKCε activation resulting in decreased insulin signaling.

### ApoC3Tg Mice Display Increased Hepatic Triglyceride Uptake

To address the underlying mechanism for the increased hepatic triglyceride content in the ApoC3Tg mice we assessed hepatic triglyceride uptake by intravenous injection of dual-labeled chylomicrons containing [^3^H]-retinyl ester (RE) and [^14^C]-triglyceride into ApoC3Tg and age-weight-matched WT littermate mice after 3 months of HFD feeding. Plasma decay of tracers in WT mice was 0.55 ± 0.15 pools/min for triglyceride and 0.19 pools/min for RE. In ApoC3Tg mice, the plasma clearance of triglyceride was reduced to 0.27 pools/min, whereas that of RE was not significantly altered (0.17 pools/min). These data suggest that the major effect of the APOC3 transgene was on the initial clearance of triglyceride.

**Fig. 4:**
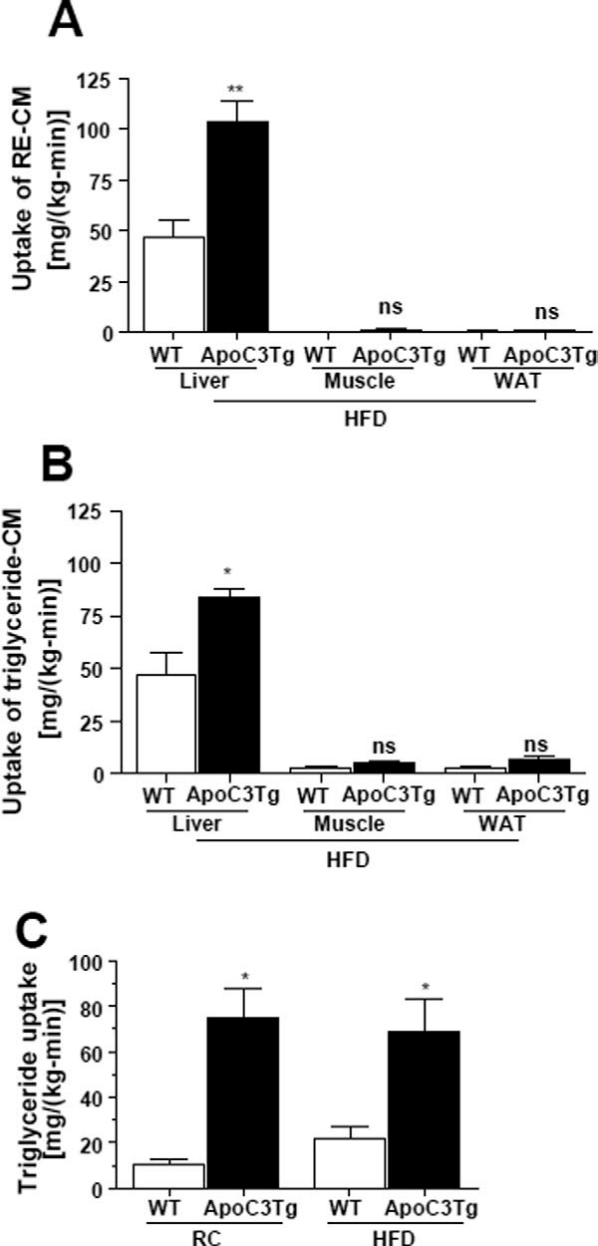
Amount of hepatic triglyceride uptake was increased in ApoC3Tg mice both on RC and HFD. Animals fed an HFD for 3 months and fasted overnight prior to experiments (A,B). After collection of blood at 0 minutes, then endogenously dual-radiolabeled chylomicron, 6 × 10^5^ dpm of [^3^H]-RE-CM and 2 × 10^5^ dpm of [^14^C]-triglyceride-CM were injected. Blood was collected at 2.5, 5, 10, and 15 minutes after injection (A,B). With a separate batch of animals fed RC or HFD for 3 months, after 6 hours fasting a bolus injection of 9 μCi of [^3^H]-triolein was intravenously administered and blood samples were collected at 2.5, 5, 7.5, 10, 15, 20, 30, and 60 minutes from tail vein (C). Tissue-specific uptake rate of (A) [^3^H]-RE-CM (B) [^14^C]-triglyceride-CM were assessed in various tissues and (C) hepatic [^3^H]-triolein uptake rate were assessed after RC and HFD. N = 4-5. *P < 0.05; **P < 0.01; ns, not significant by Student's *t* test. CM, chylomicron. RE, retinyl ester. WAT, epididymal white adipose tissue.

We then assessed the uptake of tracer into tissues with a focus on the ratio of the two labels. The uptake of both [^3^H]-RE-CM and [^14^C]-triglyceride-CM was increased by approximately twofold in the liver of ApoC3Tg mice, and the liver was the major site of TGRL uptake, compared with skeletal muscle and white adipose tissues (Fig. 4A,B). Although, in WT mice, liver triglyceride/RE uptake was 1.00 ± 0.05, indicative of greater fatty acid uptake due to lipolysis, this ratio was reduced to 0.74 ± 0.11 in the ApoC3Tg mice. Triglyceride/RE uptake was 19.4 ± 9.67 in skeletal muscle from WT mice, as would be expected due to greater fatty acid uptake after lipolysis. This ratio decreased to 6.1 ± 2.12 in the ApoC3Tg mice. APOC3 has been reported to alter TGRL metabolism either by inhibiting lipoprotein lipase (LpL) activity or interfering with receptor-mediated hepatic uptake of remnant lipoproteins.^23^ Our data suggest a block in peripheral triglyceride lipolysis consistent with LpL inhibition.

Because ApoC3Tg mice developed hepatic steatosis when fed an HFD diet but not when fed a regular chow diet, we also compared hepatic triglyceride uptake in ApoC3Tg and age-weight-matched WT littermates fed the RC and HFD. Surprisingly, we found that hepatic triglyceride uptake was increased in ApoC3Tg mice fed either the RC or HFD compared with WT mice, and that there was no further increase in hepatic triglyceride uptake in the HFD-fed ApoC3Tg mice compared with the RC-fed ApoC3Tg mice (Fig. 4C).

### Hepatic VLDL-Triglyceride Secretion Was Decreased in ApoC3Tg Mice During HFD

**Fig. 5:**
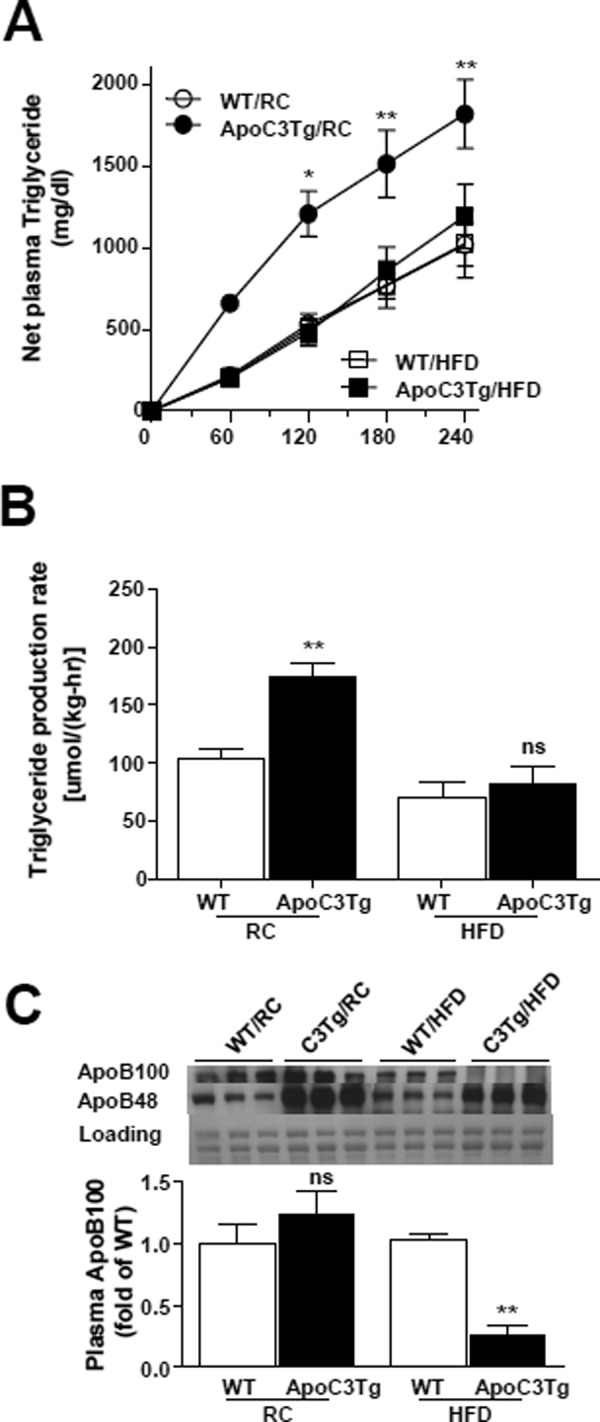
Hepatic VLDL-triglyceride secretion was decreased in ApoC3Tg mice during HFD. Animals fed an RC and HFD for 3 months and fasted overnight prior to experiments. (A) Net increase of plasma triglyceride concentration after poloxamer407 (p-407) injection from basal (0 minutes, before p-407 injection) (n = 4-6). (B) Triglyceride production rate during 4 hours after p-407 injection, expressed as micromoles of triglyceride produced per hour per kg of body weight (n = 4-6). (C) Plasma apoB determined by western blotting using 4%-12% gradient gel. The same membrane stained with Coomassie-blue was provided as loading control (n = 3, equal amount of 4 mice plasma were pooled into each sample). Data are expressed as mean ± SEM. *P < 0.05, **P < 0.01, and ***P < 0.001 by two-way ANOVA post-hoc analysis (A) and **P < 0.01 by two-tailed *t* test (B,C). ns, not significant.

Next, we assessed VLDL-triglyceride secretion in WT and ApoC3Tg mice fed either the RC or HFD by injection of the lipoprotein lipase inhibitor poloxamer407. APOC3 has been shown to stimulate VLDL synthesis and secretion in cultured cells^7^ and elevated circulating APOC3 levels correlate with VLDL production^8^ and postprandial hyperlipidemia in humans.^9^ Consistent with these findings, we observed an ≍70% increase in VLDL-triglyceride production in ApoC3Tg mice fed a RC diet (Fig. 5A,B), which was associated with marked increases in circulating apoB48 concentrations in ApoC3Tg mice (Fig. 5C). In contrast, we observed a 53% reduction in VLDL-triglyceride production rate in HFD-fed ApoC3Tg mice compared with the HFD-fed WT mice (Fig. 5B). The decreased VLDL-triglyceride production was accompanied by a marked decrease in circulating apoB100 level as well as a slight decrease in apoB48 concentrations in ApoC3Tg mice during HFD feeding (Fig. 5C). Liver microsomal triglyceride transfer protein (MTP) expression (Supporting Fig. 4) and messenger RNA (mRNA) expression of MTP (Supporting Table 2) were not significantly different between WT and ApoC3Tg mice on HFD. Thus, the greater uptake of TGRLs in the ApoC3Tg mice was not compensated by increased liver secretion of apoB-containing lipoproteins.

Other possible contributors to greater hepatic triglyceride content are *de novo* synthesis and reduced fatty acid oxidation. Interestingly, mRNA expression of hepatic sterol regulatory element binding protein-1c (SREBP-1c) and LpL were significantly higher in ApoC3Tg mice compared with WT on HFD (Supporting Table 2), indicating a possible contribution of increased hepatic lipogenesis to the increased hepatic lipid accumulation in ApoC3Tg mice during HFD feeding. There were no differences in whole-body oxygen consumption, respiratory quotients (Table 1), and liver mRNA expression of peroxisome proliferator-activated receptor-α (PPAR-α) and carnitine palmitoyltransferase 1 (CPT1), between WT and ApoC3Tg mice both on RC and HFD (Supporting Table 2), suggesting that alterations in hepatic fatty acid oxidation were not likely responsible for the observed net increases in hepatic triglyceride content observed in the HFD-fed ApoC3Tg mice.

### ApoC3Tg Mice Have Reduced Hepatic ApoB100 Expression Secondary to Postprandial Hyperinsulinemia

**Fig. 6:**
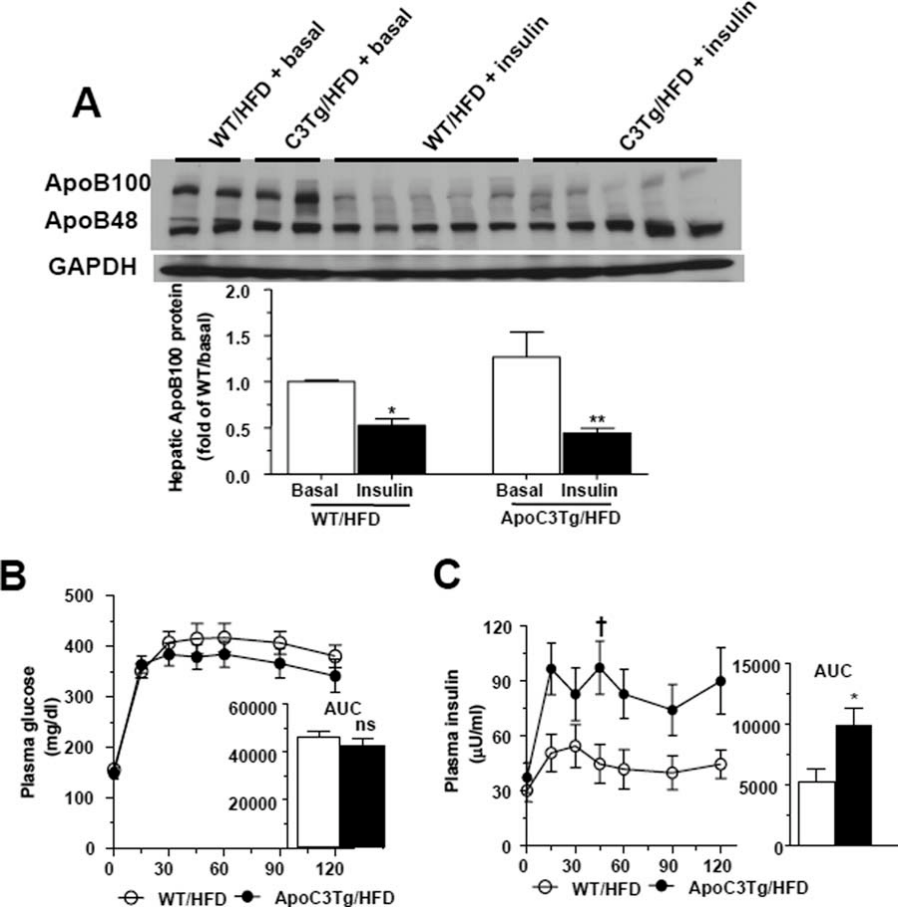
Insulin suppressed hepatic apoB100 expression and postprandial insulin secretion was increased in ApoC3Tg mice after HFD feeding. (A) Hepatic apoB100 protein expression in the liver of WT and ApoC3Tg mice from overnight fasted basal and after hyperinsulinemic-euglycemic clamp study following 2 months of HFD feeding (n = 3 for RC, n = 5 for HFD group). (B) Plasma glucose concentration and area under curve of glucose, (C) plasma insulin level and area under curve of insulin after 1 mg/kg of glucose challenge during IPGTT in WT, and ApoC3Tg mice after 6 weeks of HFD feeding (n = 7). Data are expressed as mean ± SEM. †*P* < 0.05 by two-way ANOVA analysis. *P < 0.05 and **P < 0.01 by two-tailed *t* test. ns, not significant.

Recent studies have demonstrated that insulin suppresses apoB secretion in cultured hepatocytes^24^ and *in vivo*.^25^-^27^ In order to examine the potential role of hyperinsulinemia in causing reduced hepatic apoB100 expression in ApoC3Tg mice, we measured hepatic apoB100 protein levels in HFD-fed ApoC3Tg and WT mice at the end of the hyperinsulinemic-euglycemic clamp and found that insulin rapidly suppressed hepatic apoB100 expression both in the liver of WT and ApoC3Tg mice (Fig. 6A). We next examined whether ApoC3Tg mice exhibited increased plasma insulin concentrations following an intraperitoneal glucose tolerance test. Plasma glucose concentrations were similar between HFD-fed WT and ApoC3Tg mice after glucose challenge (Fig. 5B). In contrast, ApoC3Tg mice had an ≍120% increase in peak and an ≍70% increase in the AUC of glucose-stimulated plasma insulin concentrations compared with WT mice (Fig. 5C), reflecting whole-body insulin resistance as well as increased postprandial insulin secretion in ApoC3Tg mice on HFD. Taken together, these data suggest that the increase in net hepatic triglyceride content in the HFD-fed ApoC3Tg mice can likely be attributed to both an increase in hepatic triglyceride uptake in combination with decreased hepatic VLDL secretion, due to suppression of hepatic apoB expression from chronic postprandial hyperinsulinemia.

## Discussion

Previous studies have found that whole-body insulin-mediated glucose disposal was unchanged without any ectopic lipid deposition in ApoC3Tg mice fed an RC diet.^6^, ^28^ In contrast to these findings, we found that when ApoC3Tg mice were challenged with an HFD they developed NASH associated with marked hepatic insulin resistance compared with WT littermate mice fed the same diet. These data provide important genetic verification of a recent study showing that healthy lean individuals carrying variants in the insulin response element of the APOC3 gene, leading to increased APOC3 concentrations in plasma, are predisposed to developing NAFLD associated with insulin resistance.^9^

Interestingly, we found that ApoC3Tg mice were more prone to develop hepatic steatohepatitis as reflected by the histologic NAFLD score and increased serum aspartate aminotransferase (AST), TNF-α, and INF-γ concentrations. These data are consistent with previous data demonstrating that APOC3 can cause inflammation in various cells, including endothelial cells,^10^ monocytes,^29^ and adipose tissue,^11^ and support the hypothesis that increased plasma levels of APOC3 may also make individuals more prone to develop NASH in addition to simple steatosis.^9^ However, the increased circulating cytokines were not associated with hepatic NF-κB p65, JNK phosphorylation, and ceramide content, indicating the hepatic inflammatory signals may not be the major factors for aggravating the hepatic insulin resistance in ApoC3Tg mice. In contrast, we found that the NAFLD and hepatic insulin resistance could be attributed to increased hepatocellular DAG content and activation of PKCε, which we have previously demonstrated causes decreased insulin signaling at the level of the insulin receptor kinase.^20^, ^30^ This finding is in contrast to another recent report of a PNPLA gene variant that was associated with NAFLD but was not associated with insulin resistance.^31^

Furthermore, we found that hepatic triglyceride uptake was surprisingly increased in ApoC3Tg mice fed either the RC or HFD despite the absence of hepatic steatosis when the ApoC3Tg mice were fed the RC diet. We went on to show that ApoC3Tg mice fed the HFD have reduced hepatic VLDL production, which could be attributed to decreased hepatic apoB100 expression from chronic hyperinsulinemia. Taken together, these findings suggest that increased hepatic expression of APOC3 promotes increased hepatic triglyceride uptake that is compensated for by increased hepatic VLDL production in ApoC3Tg mice fed RC. In contrast, ApoC3Tg mice fed an HFD are unable to compensate with increased VLDL production due to postprandial hyperinsulinemia, leading to suppression of hepatic apoB100 and VLDL production and to net hepatic triglyceride accumulation. In addition, we also found an ≍6-fold and ≍2-fold increase in expression of LpL and SREBP1c mRNA, respectively, in the livers of the ApoC3Tg mice following HFD, which may also have contributed to the development of hepatic steatosis in these mice. These findings may also explain the mechanism of hepatic lipid accumulation in the other mouse models of hepatic steatosis and steatohepatitis that showed severe hyperlipidemia such as apolipoprotein E (APOE)-deficient mice and LDL receptor-deficient mice.^32^-^34^

Many of the postulated effects of APOC3 on peripheral and tissue lipid metabolism were confirmed in our studies. As had been reported,^6^, ^35^ using endogenously labeled chylomicrons we confirmed that one reason for the hypertriglyceridemia in these animals is reduced plasma clearance. *In vitro* studies by these investigators found that VLDL from the APOC3 transgenic mice were lipolyzed normally by LpL but had reduced uptake into cells^6^; this latter finding is what one would expect from nonlipolyzed TGRLs that interact poorly with lipoprotein receptors. Our data showing that skeletal muscle uptake of triglyceride is much more impaired than its uptake of RE is more consistent with APOC3 functioning as an inhibitor of lipolysis. In addition, the observations that APOC3 overexpression leads to hypertriglyceridemia in APOE knockout mice^36^ and causes less hypertriglyceridemia in apoB48 only mice that are more dependent on non-LDL receptor-mediated lipid uptake^37^ are consistent with LpL inhibition.

In summary, we found that transgenic mice with hepatic overexpression of human APOC3 were more prone to develop hepatic steatosis associated with hepatic insulin resistance compared with WT littermates fed the same HFD diet. This study provides strong genetic evidence in support of the hypothesis that increased plasma APOC3 concentrations predispose lean individuals to NAFLD associated with hepatic insulin resistance.^9^ Importantly, the development of NAFLD associated with hepatic insulin resistance in response to dietary alterations exemplifies how gene-environment interactions contribute to the pathogenesis of complex phenotypes.

## Abbreviations

apoB: apolipoprotein B
APOC3: apolipoprotein CIII
ApoC3Tg: transgenic mice with hepatic overexpression of human APOC3
APOE: apolipoprotein E
AUC: area under curve
CM: chylomicron
DAG: diacylglycerol
EGP: endogenous glucose production
HFD: high-fat diet
IPGTT: intraperitoneal glucose tolerance test
JNK: c-Jun N-terminal kinase
LpL: lipoprotein lipase
MTP: microsomal triglyceride transfer protein
NAFLD: nonalcoholic fatty liver disease
NASH: nonalcoholic steatohepatitis
NAS: NAFLD activity score
NF-κB: nuclear factor kappaB
PKCε: protein kinase C-ε
PPAR-α: peroxisome proliferator-activated receptor
RC: regular chow
RE: retinyl ester
SREBP-1c: sterol regulatory element binding protein-1c
TGRLs: triglyceride rich lipoproteins
TNF-α: tumor necrosis factor-alpha
VLDL: very low density lipoproteins
WT: wildtype littermates
