# Supplementary figures and images for: Apolipoprotein CIII Overexpressing Mice Are Predisposed to Diet-Induced Hepatic Steatosis and Hepatic Insulin Resistance
Source: Hepatology. 2011 Aug 19;54(5):1650–60. doi: 10.1002/hep.24571 (PMC3205235; doi:10.1002/hep.24571)

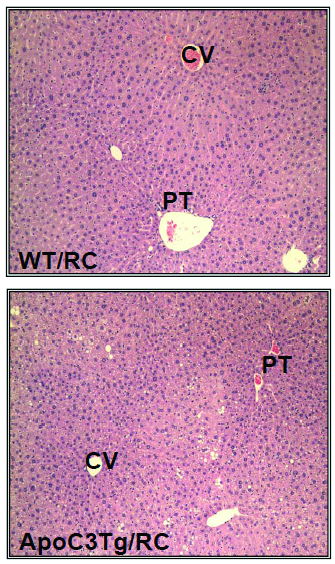

Supplement: Supplementary file 1 [file hep0054-1650-SD1.tif]

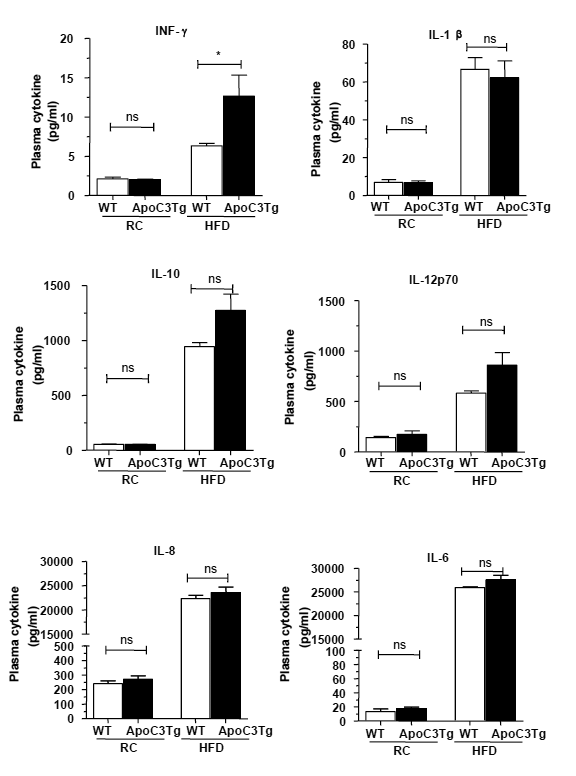

Supplement: Supplementary file 2 [file hep0054-1650-SD2.tif]

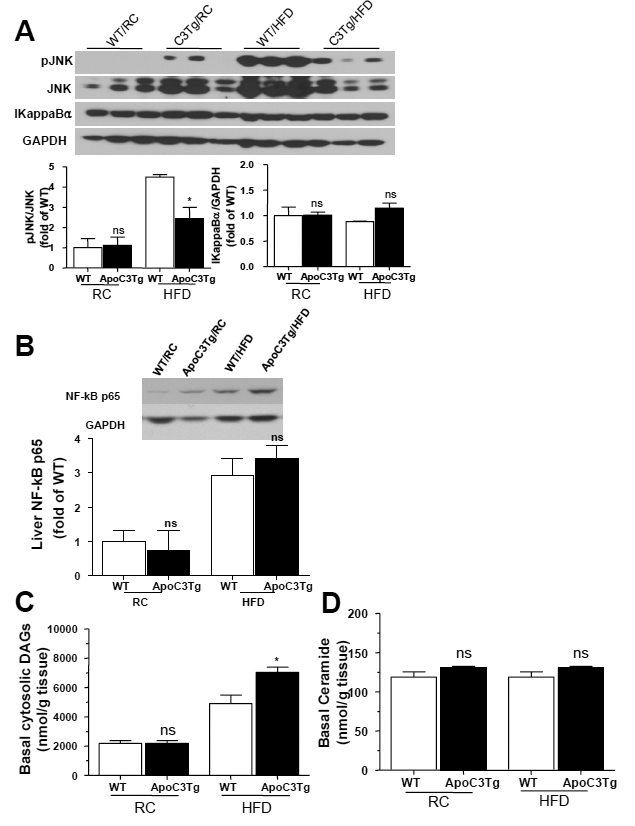

Supplement: Supplementary file 3 [file hep0054-1650-SD3.tif]

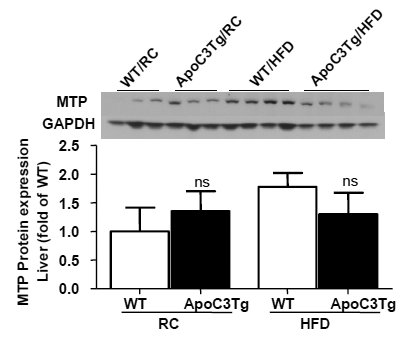

Supplement: Supplementary file 4 [file hep0054-1650-SD4.tif]

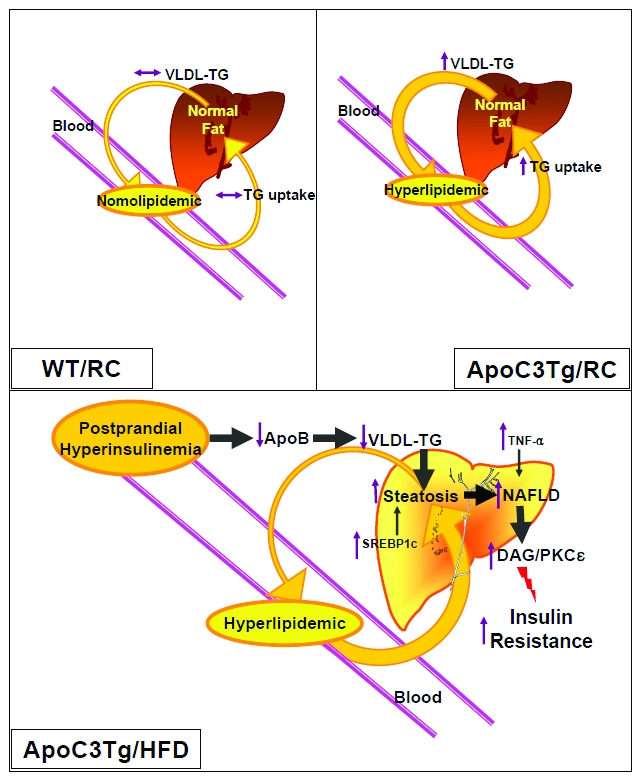

Supplement: Supplementary file 5 [file hep0054-1650-SD5.tif]
