# Supplementary material for: Apolipoprotein CIII Overexpressing Mice Are Predisposed to Diet-Induced Hepatic Steatosis and Hepatic Insulin Resistance
Source: Hepatology. 2011 Aug 19;54(5):1650–60. doi: 10.1002/hep.24571 (PMC3205235; doi:10.1002/hep.24571)
Supplement: Supplementary file 6 [file hep0054-1650-SD6.doc]

**Supporting Table 1.** Hyperinsulinemic-Euglycemic Clamp Data

|  | Wild Type (n=6 ) | C3Tg (n=7 ) | ***P value*** |
| --- | --- | --- | --- |
| **Body Weight** | 36.4 ± 0.6 | 38.7 ± 1.6 | NS |
| **Basal Plasma Glucose (mg/dL)** | 169.8 ± 15.4 | 154.7 ± 11.6 | NS |
| **Clamp Plasma Glucose (mg/dL)** | 116.5 ± 2.2 | 116.3 ± 3.2 | NS |
| **Basal EGP [mg/(kg-min)]** | 11.9 ± 1.0 | 10.9 ± 0.7 | NS |
| **Clamp EGP [mg/(kg-min)]** | 8.6 ± 0.9 | 12.1 ± 1.2 | <0.05 |
| **GINF [mg/(kg-min)]** | 23.3 ± 1.7 | 15.6 ± 1.7 | <0.01 |
| **Rd [mg/(kg-min)]** | 31.9 ± 1.3 | 27.7 ± 0.7 | <0.05 |
| **Basal Plasma Insulin (µU/mL)** | 23.9 ± 3.1 | 28.6 ± 3.8 | NS |
| **Clamp Plasma Insulin (µU/mL)** | 176.4 ± 22.0 | 198.4 ± 30.0 | NS |

Key variables pertaining to the 4.5 mU/kg-min hyperinsulinemic-euglycemic clamp experiment in WT and ApoC3Tg mice fed a HFD for 2 month and fasted 14 h prior to experiments. All infusions were delivered via a jugular venous catheter and blood sampled from a distal tail incision. Data are expressed as mean values ± SEM. *P-value* evaluated by unpaired Student’s t-test. NS, not significant; EGP, endogenous glucose production; GINF, glucose infusion rate; Rd, whole body glucose disposal rate.

**Supporting Table 2. Liver mRNA expression and primers.**

|  | **RC** | | **HFD** | | *P-value* | *P-value* |
| --- | --- | --- | --- | --- | --- | --- |
| **Gene** | WT  (n=5) | C3Tg  (n=4) | WT  (n=4) | C3Tg  (n=6) | WT vs. C3Tg  On RC | WT vs. C3Tg  On HFD |
| **SREBP1c** | 1.0 ± 0.38 | 1.7 ± 0.17 | 2.0 ± 0.48 | 3.3 ± 0.27 | NS | <0.05 |
| **SCD1** | 1.0 ± 0.56 | 1.2 ± 0.40 | 2.2 ± 0.79 | 3.9 ± 0.45 | NS | 0.07 |
| **FATP** | 1.0 ± 0.41 | 1.3 ± 0.51 | 19.6 ± 5.11 | 20.8 ± 1.96 | NS | NS |
| **CD36** | 1.0 ± 0.22 | 1.5 ± 0.64 | 13.7 ± 2.72 | 19.7 ± 2.86 | NS | NS |
| **MTP** | 1.0 ± 0.23 | 1.4 ± 0.26 | 2.2 ± 0.37 | 2.3 ± 0.15 | NS | NS |
| **LPL** | 1.0 ± 0.27 | 0.9 ± 0.20 | 1.8 ± 0.48 | 6.9 ± 0.99 | NS | <0.01 |
| **DGAT2** | 1.0 ± 0.21 | 1.2 ± 0.36 | 2.7 ± 0.37 | 3.1 ± 0.19 | NS | NS |
| **ChREBP** | 1.0 ± 0.45 | 2.4 ± 1.09 | 5.7 ± 1.37 | 6.9 ± 0.68 | NS | NS |
| **HL** | 1.0 ± 0.20 | 1.5 ± 0.28 | 3.8 ± 0.39 | 3.4 ± 0.08 | NS | NS |
|  |  |  |  |  |  |  |
| **LDLR** | 1.0 ± 0.44 | 1.8 ± 0.36 | 2.7 ± 0.75 | 2.9 ± 0.22 | NS | NS |
| **LRP1** | 1.0 ± 0.54 | 2.2 ± 0.88 | 4.4 ± 1.37 | 5.2 ± 0.49 | NS | NS |
| **LSR** | 1.0 ± 0.21 | 1.2 ± 0.38 | 1.5 ± 0.28 | 2.1 ± 0.22 | NS | NS |
| **ApoE** | 1.0 ± 0.19 | 1.2 ± 0.17 | 3.5 ± 0.48 | 3.5 ± 0.20 | NS | NS |
| **FAS** | 1.0 ± 0.67 | 2.1 ± 0.96 | 0.7 ± 0.21 | 1.3 ± 0.23 | NS | NS |
| **Cideb** | 1.0 0.17 | 1.2 ± 0.22 | 2.6 ± 0.33 | 2.9 ± 0.21 | NS | NS |
| **PPARα** | 1.0 0.24 | 1.6 ± 0.19 | 3.8 ± 0.57 | 4.6 ± 0.19 | NS | NS |
| **CPT1** | 1.0 0.19 | 1.4 ± 0.34 | 6.9 ± 0.90 | 9.7 ± 1.8 | NS | NS |

| **Gene** | Forward primer  (5’ to 3’) | Reverse primer  (5’ to 3’) | **Gene** | Forward primer  (5’ to 3’) | Reverse primer  (5’ to 3’) |
| --- | --- | --- | --- | --- | --- |
| **SREBP1c** | CAGCTCAGAGCCGTGGTGA | TTGATAGAAGACCGGTAGCGC | **HL** | GACTGGATCTCCCTGGCATA | AGGTGAACTTTGCTCCGAGA |
| **SCD1** | AGATCTCCAGTTCTTACACGACCAC | GACGGATGTCTTCTTCCAGGTG | **LDLR** | CTGGTGACCGAAAACATCCAGT | AATCAACCCAATAGAGACGGCC |
| **FATP** | GCGTTTCGATGGTTATGT | TTGAGTTAGGGTCCAACTG | **LRP1** | GTGTATGACGAGAGCATCCAGCTAG | AGGAGCGAGTCGTCTCTGATGTT |
| **CD36** | ATTGGTCAAGCCAGCT | TGTAGGCTCATCCACTAC | **LSR** | CAGGAGAATCACCATCACAGGAA | AGTAATACACTCCACTGTCTCCCCAG |
| **MTP** | GGGCTGGAGTTCATCTCCAC | GCCTTGTCCATCTGCATGC | **APOE** | AGGTCCAGGAAGAGCTGCAG | CCTTTACTTCCGTCATAGTGTCCTC |
| **LPL** | TGGAGAAGCCATCCGTGTG | TCATGCGAGCACTTCACCAG | **FAS** | GCTGCGGAAACTTCAGGAAAT | AGAGACGTGTCACTCCTGGACT |
| **DGAT2** | ACTCTGGAGGTTGGCACCAT | GGGTGTGGCTCAGGAGGAT | **Cideb** | CTGGAACTCAGCTCCTCCAC | CAGCAGCTGGAAGAAGTCCT |
| **ChREBP** | ACCTGTCTCCCCCTCAAACT | TGTCTTCTGAAGCGTGGTTG | **PPARα** | GCAGCTCGTACAGGTCATCA | CTCTTCATCCCCAAGCGTAG |

WT and ApoC3Tg mice fed a RC and HFD for 3 month and fasted overnight prior to experiments. Liver tissue total RNA was isolated using Trizol by manufacturer instructions (Invitrogen), and cDNA was reverse transcribed from 1µg total RNA. Quantitative real-time PCR was performed using Mx7500 pultiplex PCR system with SYBR Green PCR core reagents (Applied Biosystems). Relative quantification of gene expression was performed using the standard curve method with cyclophilin as the housekeeping gene. Data are normalized by average of WT on RC, and expressed as mean values ± SEM. *P-value* evaluated by unpaired Student’s t-test compared to WT versus C3Tg group. N=4-6. Sterol regulatory element binding protein-1c (SREBP-1c), stearoyl-CoA desaturase-1 (SCD1), fatty acid transport protein (FATP), microsomal triglyceride transfer protein (MTP), lipoprotein lipase (LPL), diacylglycerol O-acyltransferase 2 (DGAT2), carbohydrate response element binding protein (ChREBP), hepatic lipase (HL), low density lipoprotein receptor (LDLR), low density lipoprotein receptor-related protein 1 (LRP1), lipolysis stimulated lipoprotein receptor (LSR), apolipoprotein E (ApoE), fatty acid synthase (FAS), peroxisome proliferator-activated receptor alpha (PPARα), carnitine palmitoyltransferase 1 (CPT1). NS, not significant.

**Supporting Figure 1, related to figure 1. Liver histology for WT and ApoC3Tg mice on regular chow.** Liver sections for histology were obtained after overnight fasting, fixed in 10% formalin, and stained with hematoxylin-eosin and examined ×100 magnification.

**Supporting Figure 2, related to figure 1. Proinflammatory cytokine array.** Animals fed RC or HFD for 3 months. Plasma samples were taken from the tail vein after an overnight fast, and pooled 4 mice for each sample (n=3 from total 12 mice per group). Mouse cytokine array (RayBio, CL, USA), was conducted as described in the manufacturers description. The values of all cytokines in WT and ApoC3Tg mice were significantly increased during HFD feeding, compared to RC group (P<0.05). *P<0.05; ns, not significant by Student’s t-test.

**Supporting Figure 3, related to figure 3. Liver ceramide, phosphorylation of JNK, IKappaBα and NF-κb p65 expression in the liver from WT and ApoC3Tg mice.** Animals fed RC or HFD for 2-3 months. Liver samples were taken from WT and C3Tg mice after an overnight fast, and JNK phosphorylation and IKappaBα (A) and NF-κB p65 protein expression (B), Basal cytosolic DAGs (C) and ceramides content (D) were determined in the liver from WT and ApoC3Tg mice. Total ceramide were expressed as the sum of individual species. N=3-4 per group for panel A and B and n=4-6 for panel C and D. *P<0.05; ns, not significant by Student’s t-test.

**Supporting Figure 4, related to figure 6. Basal hepatic MTP protein expression in WT and ApoC3Tg mice.** Animals fed regular chow or HFD for 3 months. Liver samples were taken from WT and ApoC3Tg mice after an overnight fast, and MTP protein expression were determined in the liver from WT and ApoC3Tg mice. (n=5 for RC group, n=8 for HFD group). Data are expressed as mean ± SEM. ns, not significant.

**Supporting Figure 5, Mechanism of hepatic triglyceride accumulation and hepatic insulin resistance in ApoC3Tg mice.** On regular chow, the lipid uptake and production in the liver of ApoC3Tg mice characterized as high turn-over status, revealed by both liver triglyceride uptake and VLDL-triglyceride output are increased (upper right), compared to those of WT mice, and both result in normal net lipid accumulation in liver. In contrast, on high fat diet (bottom), the increased postprandial insulin concentration in ApoC3Tg mice suppress the VLDL-triglyceride secretion without altering hepatic lipid uptake, and these lead to increase of hepatic lipid accumulation and hepatic insulin resistance. Thus, the increase in net hepatic triglyceride content in the HFD ApoC3Tg mice can likely be attributed to both an increase in hepatic triglyceride uptake in combination with decreased hepatic VLDL secretion, due to suppression of hepatic Apo B expression from chronic postprandial hyperinsulinemia. SREBP1c mediated lipid synthesis and increased serum cytokines may contribute to the lipid accumulation and hepatic insulin resistance as cofactors while the increased diacylglycerol and PKCε are likely to be major factors for the hepatic insulin resistance in C3Tg mice during high-fat diet feeding.
